# Morphine promotes cancer stem cell properties, contributing to chemoresistance in breast cancer

**DOI:** 10.18632/oncotarget.2894

**Published:** 2015-02-20

**Authors:** Dong-Ge Niu, Fei Peng, Wei Zhang, Zhong Guan, Hai-Dong Zhao, Jing-Lin Li, Kai-Li Wang, Ting-Ting Li, Yan Zhang, Fei-Meng Zheng, Fan Xu, Qian-Ni Han, Peng Gao, Qing-Ping Wen, Quentin Liu

**Affiliations:** ^1^ Institute of Cancer Stem Cell, Dalian Medical University, Dalian 116044, China; ^2^ Sun Yat-sen University Cancer Center, State Key Laboratory of Oncology in South China, Collaborative Innovation Center of Cancer Medicine, Guangzhou 510060, China; ^3^ Department of Anesthesia, The First Affiliated Hospital, Dalian Medical University, Dalian 116044, China; ^4^ Department of Otorhinolaryngology, Sun Yat-sen Memorial Hospital, Sun Yat-sen University, Guangzhou 510120, China; ^5^ Department of Breast Surgery, The Second Affiliated Hospital of Dalian Medical University, Dalian 116023, China; ^6^ Department of Clinical Pharmacology, College of Pharmacy, Dalian Medical University, Dalian 116044, China

**Keywords:** Morphine, cancer stem cell, chemoresistance, nalmefene, epithelial to mesenchymal transition

## Abstract

Morphine is an opioid analgesic drug commonly used for pain relief in cancer patients. Here, we report that morphine enhances the mammosphere forming capacity and increases the expression of stemness-related transcription factors Oct4, Sox2 and Nanog. Treatment with morphine leads to enrichment of a side population fraction in MCF-7 cells and the CD44^+^/CD24^−/low^ population in BT549 cells. Consistently, morphine activates Wnt/β-catenin signaling to induce epithelial to mesenchymal transition and promotes metastasis. Moreover, morphine decreases the sensitivity of traditional anti-cancer drugs in breast cancer cells. Nalmefene, an antagonist of morphine, reverses morphine-induced cancer stem cell properties and chemoresistance in breast cancer. In addition, nalmefene abolishes morphine enhancing tumorigenesis in a NOD/SCID mouse model. In conclusion, our findings demonstrate that morphine contributes to chemoresistance via expanding the population of cancer stem cells and promotes tumor growth, thereby revealing a novel role of morphine and providing some new guides in clinical use of morphine.

## INTRODUCTION

Breast cancer is the most common cancer in women worldwide [1]. Breast cancer contains a subpopulation of self-renewing cells that resemble mammary stem cells. Breast cancer stem cells (BCSCs) first isolated by surface marker CD44^+^/CD24^−/low^ possess the characteristic of unlimited self-renewal and are able to generate differentiated descendants [2]. Inoculation of small numbers of CD44^+^/CD24^−/low^ breast cancer cells in NOD/SCID mice can recapitulate the phenotypic heterogeneity of the parent tumor, whereas cells lacking the CD44^+^/CD24^−/low^ marker have a greatly reduced tumor-forming capacity [3]. BCSCs are more resistant to traditional chemotherapy than non-BCSCs [4]. The chemoresistance of BCSCs is tightly regulated by the following mechanisms: 1. the elevation of the ABC transporters that function to efflux chemotherapeutic drugs [5]; 2. the differentiation ability of CSCs to regenerate heterogeneous cancer cells [6, 7]; 3. the enrichment of CSCs during therapeutic intervention may contribute to the genesis of CSCs and increase the fraction of CSCs [8]. Thus targeting CSCs is the current challenge for the development of anti-cancer treatment. However, the molecular mechanisms by which CSCs contribute to drug resistance remain to be determined.

Epithelial to mesenchymal transition (EMT) is a transdifferentiation program that is initially recognized as a key step for morphogenesis during embryonic development [9]. Recent advance indicates that EMT plays an important role during the development of drug resistance in breast cancer. Elevated expression of E-cadherin enhances the sensitivity of EGFR kinase inhibitors and the drug resistant cells are found to be with more mesenchymal-like characteristic [10]. Moreover, EMT is also involved in paclitaxel-resistance of breast cancer cells. Accordingly the resistant cells display EMT features and increase invasion and migration [11].

Opioids, such as morphine, are the most potent analgesics, which have been extensively used for anesthetic pre-medication and management of cancer pain with cancer metastasis. Currently, both morphine and anticancer drugs have been simultaneously given to patients, especially those patients with cancer metastasis. However, emerging evidence showed that morphine had extra analgesic effects that appeared to alter tumor progression by activating non-classical opioid receptor signaling. Morphine induces phosphorylation of epidermal growth factor receptor (EGFR) via opioid receptors, promotes cell proliferation and increases cell invasion [12]. Morphine also activates MAPK/ERK by phosphorylation via PTX-sensitive GPCRs and NO, which leads to the promotion of tumor growth in breast cancer [13]. In addition, morphine promotes breast cancer cell migration and invasion by increasing the expression of NET1 [14]. Until now, little attention has been paid to the development of drug resistance during application of morphine.

Therefore, the key aim of this study was to address whether morphine played important roles in regulating cancer stem cell properties, which were closely correlated with the chemoresistance of cancer cells and tumor malignancy. In the present study, we found that the application of morphine in MCF-7 and BT549 cells enriched cancer stem cell populations and contributed to the development of chemoresistance. We also found that morphine enhanced the tumorigenicity of breast cancer cells, which could be blocked by nalmefene in NOD/SCID mouse model. Our study also suggested that morphine contributed to the acquired chemoresistance in breast cancer. These results revealed the possible side effects of morphine in cancer development and chemoresistance, which may provide some guides in the clinical use of morphine.

## RESULTS

### Morphine promotes cancer stem cell properties

**Figure 1:**
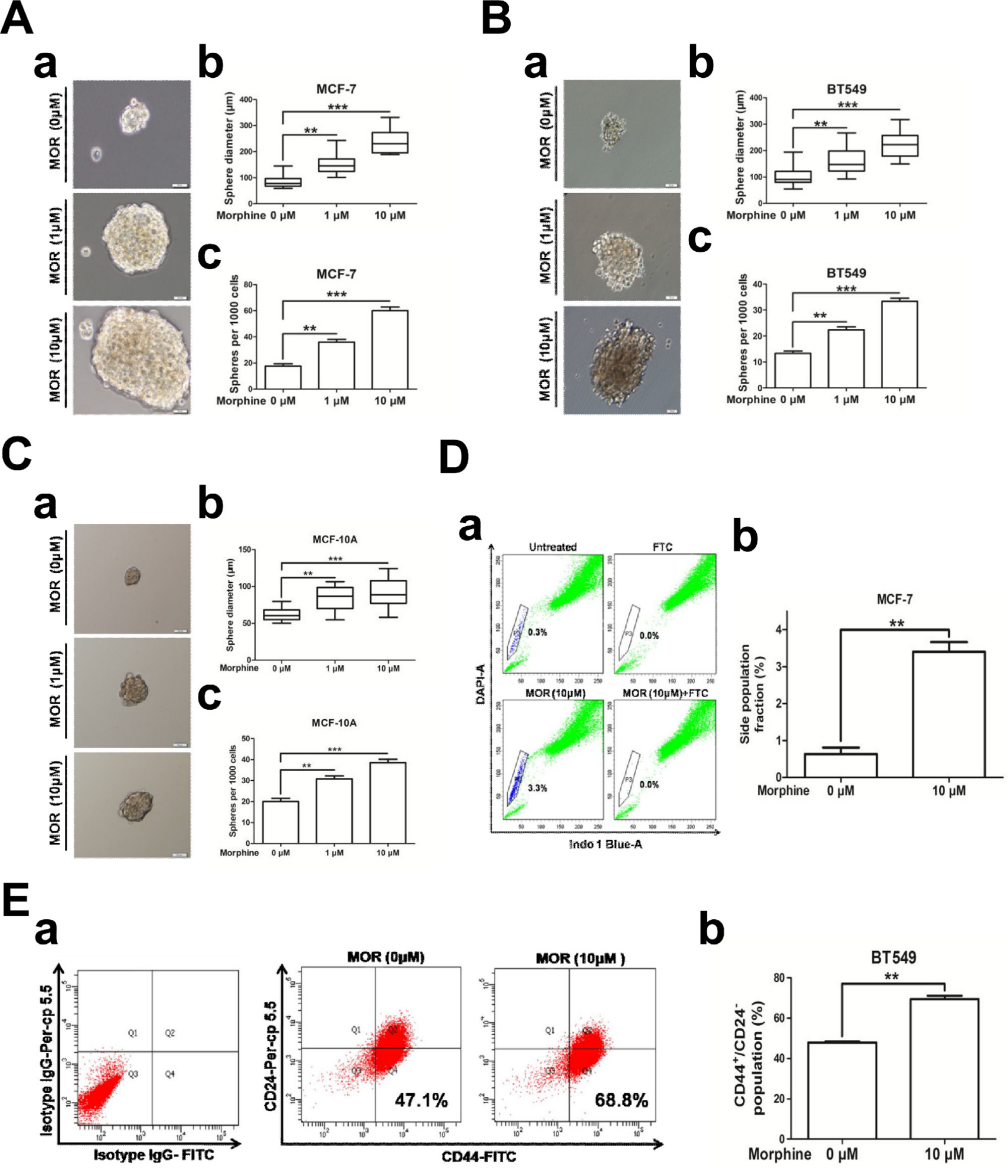
Morphine promotes cancer stem cell properties **(A–C)** a Representative pictures of mammospheres formed by MCF-7, BT549 and MCF-10A cells after treating with morphine (0, 1, 10 μM) for 14 days, respectively (Scale bars, 50 μm). b–c Bar diagrams showed the diameter and number of mammospheres (spheres > 50 μm). **(D)** a Representative Hoechst 33342 dye staining profile of morphine untreated MCF-7 cells in the absence or presence of FTC (up) and MCF-7 cells treated with 10 μM morphine for 14 days in the absence or presence of FTC (down). b Bar diagram showed the percentage of side population cells in morphine treated and untreated MCF-7 cells. **(E)** a Provided pictures were representative flow cytometry dot plots of CD44 and CD24 expression of BT549 cells treated with morphine (0, 10 μM) for 14 days. b Bar diagram showed the population of CD44^+^/CD24^−^ cells changed in morphine untreated and treated MCF-7 cells as described left. *P < 0.05, **P < 0.01, ***P < 0.001. Error bars represent mean ± SD of triplicates.

To investigate the potential role of morphine in promoting cancer stem cell properties, we chose MCF-7 and BT549 cell lines which included relatively low population of cancer stem cells [20, 21]. We applied mammosphere formation assay and side population assay in breast cells. Mammosphere formation assay has been used as a surrogate reporter of stem cell activity in the mammary gland [22] and cancer stem cell activity [23]. We treated MCF-7, BT549 and MCF-10A cells with morphine (0, 1, 10 μM), and performed a sphere formation assay [24, 25]. Results showed that morphine significantly increased the sphere size and number in both MCF-7 cells (Figure 1A) and BT549 cells (Figure 1B). Similar results were observed in non-transforming mammary epithelial cell MCF-10A (Figure 1C). For side population assay, we pretreated MCF-7 cells with morphine (10 μM) for 14 days, passaged cells with fresh medium containing morphine every 2 days and performed side population assay coupled with flow cytometry analysis. Our results showed that side population fraction increased from 0.63 ± 0.30% to 3.4 ± 0.15% compared with the untreated group in MCF-7 cells (Figure 1D). Since CD44^+^/CD24^−^ were widely used as a cell surface marker of CSCs, we next detected the expression of CD44^+^/CD24^−^ in breast cancer cells treated with morphine (10 μM) and found that the CD44^+^/CD24^−^ proportion were increased from 47.87 ± 1.01% to 68.8 ± 2.68% in BT549 cells (Figure 1E). These results demonstrate that morphine increases the tumorsphere-forming ability and enriches cancer stem cells, indicating that morphine enhances the self-renewal capacity in breast cancer cells and normal mammary epithelial cells.

### Morphine increases the expression of Sox2, Oct4 and Nanog

**Figure 2:**
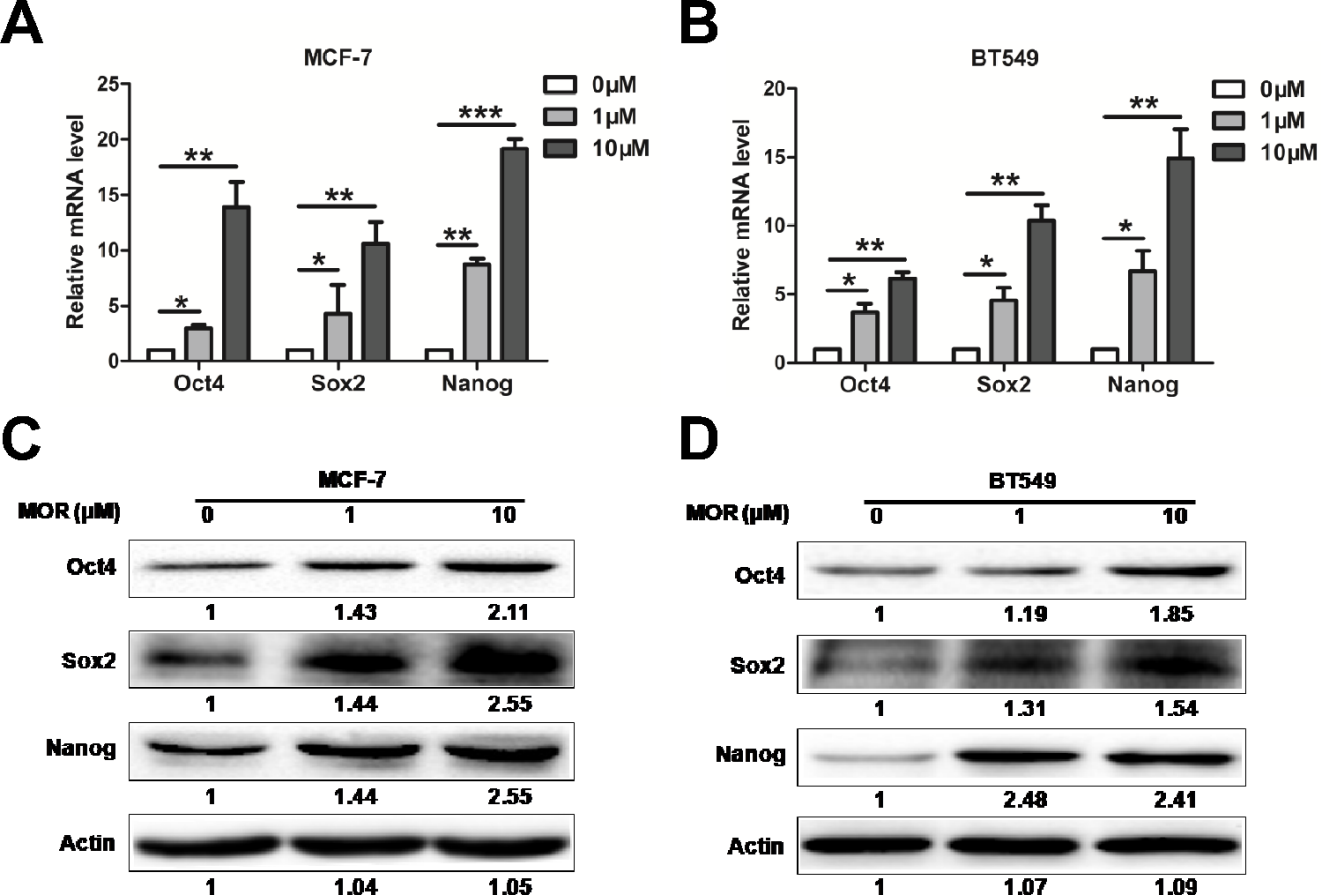
Morphine increases the expression of Sox2, Oct4 and Nanog **(A–B)** The mRNA levels of Sox2, Oct4 and Nanog in MCF-7 and BT549 cells were measured by Q-PCR after treating with morphine (0, 1, 10 μM) for 4 days. *P < 0.05, **P < 0.01, ***P < 0.001. Error bars represent mean ± SD of triplicates. **(C–D)** MCF-7 and BT549 cells were treated with morphine (0, 1, 10 μM) for 4 days. Sox2, Oct4 and Nanog protein levels of cell lysates were detected by western blotting.

Sox2, Oct4 and Nanog are transcription factors that play key roles in maintaining the pluripotency of embryonic stem cells [26–28]. To explore the underlying mechanism by which morphine promotes the CSC properties of breast cancer cells, we examined the expression of Sox2, Oct4 and Nanog following morphine treatment. Firstly, we examined the mRNA levels of Oct4, Sox2 and Nanog in MCF-7 and BT549 cells treated with morphine by Q-PCR. Morphine significantly increased the mRNA levels of Oct4, Sox2 and Nanog in both MCF-7 and BT549 cells. In comparison to untreated controls, the mRNA levels of Oct4, Sox2 and Nanog were increased respectively by 13.08 ± 2.29, 10.57 ± 1.42 and 19.18 ± 0.85 folds in MCF-7 cells (Figure 2A), while 6.15 ± 0.61, 10.37 ± 0.91 and 14.92 ± 1.47 folds in BT549 cells (Figure 2B). Consistently, western blot assay showed that morphine dose dependent increased the protein levels of Oct4, Sox2, Nanog in MCF-7 (Figure 2C) and BT549 cells (Figure 2D). These data suggest that morphine may promote cancer stem cell properties by up-regulating Oct4, Sox2 and Nanog.

### Morphine promotes EMT and metastasis

**Figure 3:**
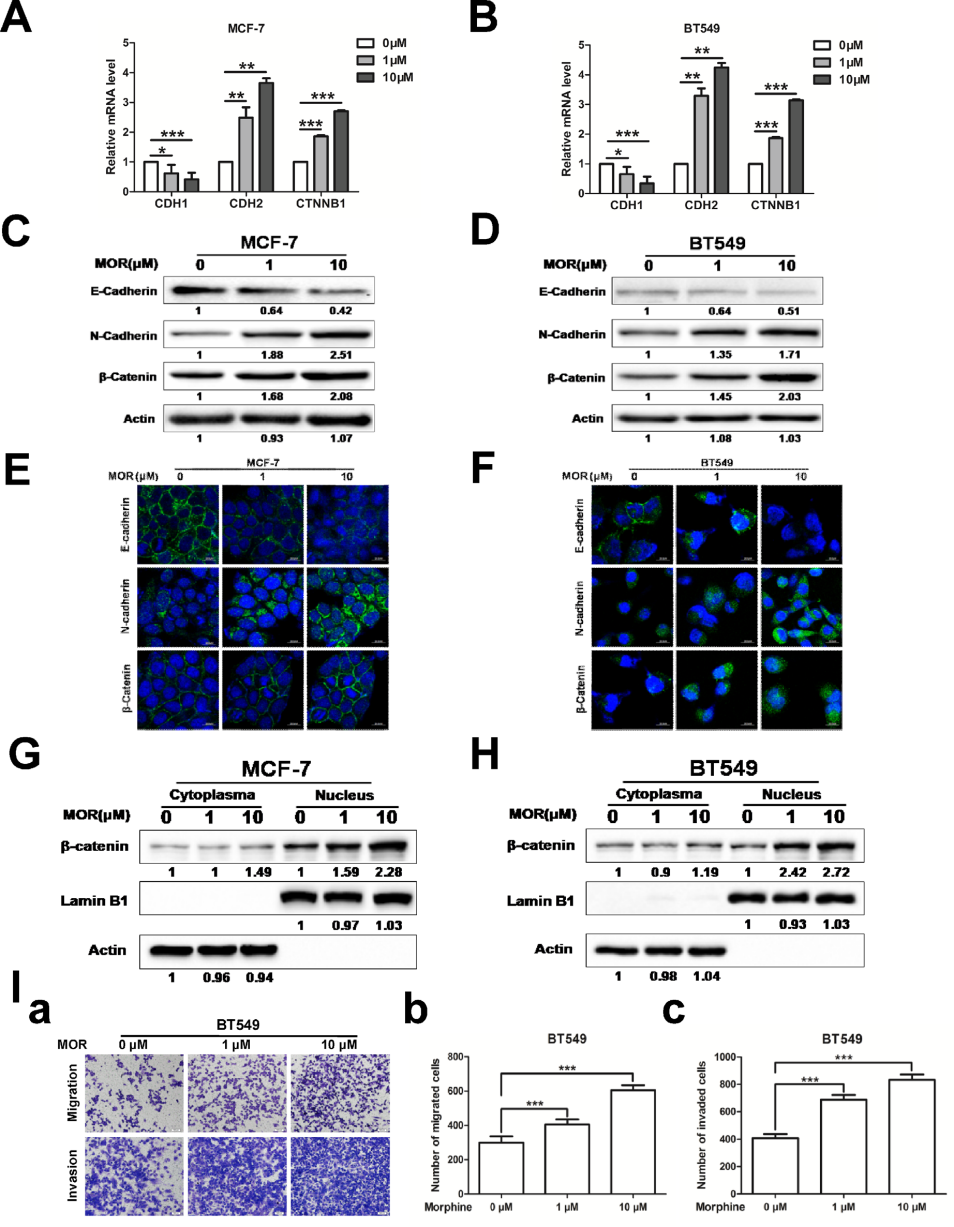
Morphine promotes EMT and metastasis MCF-7 and BT549 cells were treated with morphine (0, 1, 10 μM) for 4 days. **(A–B)** The mRNA levels of CDH1, CDH2 and CTNNB1 were measured by Q-PCR. *P < 0.05, **P < 0.01, ***P < 0.001. Error bars represent mean ± SD of triplicates. **(C–D)** E-cadherin, N-cadherin and β-catenin levels of cell lysates were measured by western blotting analysis. **(E–F)** Immunofluorescence was performed using FITC-labeled phalloidin, E-cadherin, N-cadherin, β-catenin. Nuclei were stained with DAPI (Scale bar, 20 μm). **(G–H)** Cells were subjected to nuclear/cytoplasmic protein isolation. The expressions of β-catenin in nucleus and cytoplasm were determined by western blotting. Lamin B1 and β-actin were taken as nuclear and cytoplasmic control respectively. **(I)** a Representative images of migrated and invaded cells are shown. b–c Bar diagrams showed the results of migrated and invaded cells untreated and treated with morphine in BT549 cells as described. *P < 0.05, **P < 0.01, ***P < 0.001. Error bars represent mean ± SD of triplicates.

EMT is often accompanied by an increase of cancer stem cells [29, 30]. We next examined whether morphine was associated with the induction of EMT and tumor metastasis. We assessed the expression of epithelial marker E-cadherin and mesenchymal marker N-cadherin in MCF-7 and BT549 cells using Q-PCR, western blot and immunofluorence staining. Morphine decreased the mRNA level of CDH1 but increased the mRNA levels of CDH2 and CTNNB1 in both MCF-7 (Figure 3A) and BT549 (Figure 3B) cells. Consistently, morphine decreased the expression of E-cadherin but increased the expression of N-cadherin and β-catenin in MCF-7 (Figure 3C) and BT549 (Figure 3D) cells. Moreover, the immunofluorence staining results also showed that morphine decreased the expression of E-cadherin while increased the expression of N-cadherin and β-catenin in both MCF-7 (Figure 3E) and BT549 (Figure 3F) cells. Furthermore, we investigated the expression of β-catenin in cytoplasma and nucleus individually for Wnt/β-catenin activation. Results showed that β-catenin increased its expression in nucleus but not in cytoplasma in both MCF-7 (Figure 3G) and BT549 (Figure 3H) cells. Meanwhile, as EMT is a key process in cancer metastasis [31], we examined the role of morphine in tumor metastasis by transwell assay. Results showed that morphine could significantly enhance cell migration and invasion abilities in BT549 cell (Figure 3I). These results suggest that morphine promotes EMT and metastasis in breast cancer.

### Morphine induces resistance to doxorubicin and paclitaxel

**Figure 4:**
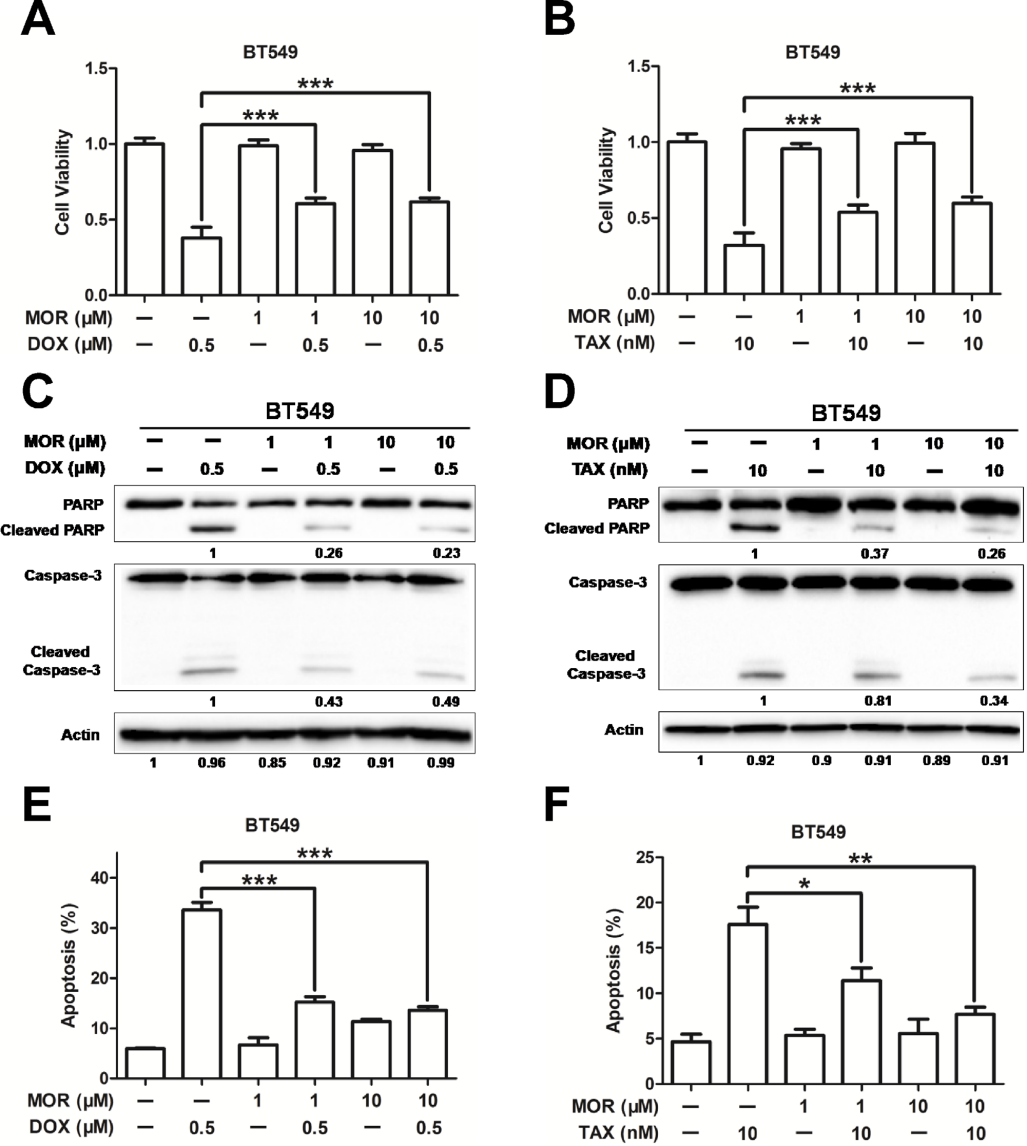
Morphine induces resistance to doxorubicin and paclitaxel BT549 cells were pretreated with morphine at the concentraton of 0, 1, 10 μM for 4 days and combination with doxorubicin (0.5 μM) or paclitaxel (10 nM) for another 2 days. **(A–B)** Cell viabilities were measured by MTT assay. **(C–D)** Cleavaged caspase-3 and PARP were detected by western blotting. **(E–F)** Cell apoptosis was measured by Annexin V/PI co-staining assay. *P < 0.05, **P < 0.01, ***P < 0.001. Error bars represent mean ± SD of triplicates.

The promotion of the side population proportion suggests that morphine may mediate chemoresistance. To demonstrate this hypothesis, we firstly treated MCF-7 and BT549 cells with increasing concentrations of doxorubicin or paclitaxel and analyzed cell viability by MTT assay after 24 and 48 hours. Both doxorubicin and paclitaxel decreased cell proliferation in a dosage dependent manner of MCF-7 and BT549 cells (Figure S1). We next investigated whether morphine was able to reduce the sensitivity of BT549 cells to chemotherapy. Cells were pretreated with morphine for 4 days, followed by incubated with doxorubicin (0.5 μM) or paclitaxel (10 nM) for another 2 days. Our results showed that morphine (1 μM or 10 μM) alone did not apparently alter the cell viability, but significantly abolished the loss of cell viability induced by doxorubicin (Figure 4A) or paclitaxel (Figure 4B). We further analyzed the effect of morphine on doxorubicin or paclitaxel induced apoptosis in breast cancer cells by assessing cleaved PARP and caspase-3 by western blot. Our results showed that doxorubicin (Figure 4C) or paclitaxel (Figure 4D) induced the cleavage of PARP and caspase-3, but were apparently recovered by morphine. Moreover, treatment of BT549 cells with doxorubicin (Figure 4E) or paclitaxel (Figure 4F) resulted in cell apoptosis, which was also apparently recovered by morphine. Taken together, these findings indicate that morphine induces resistance of doxorubicin and paclitaxel in breast cancer cells.

### Nalmefene reverses morphine-induced cancer stem cell properties and chemoresistance

**Figure 5:**
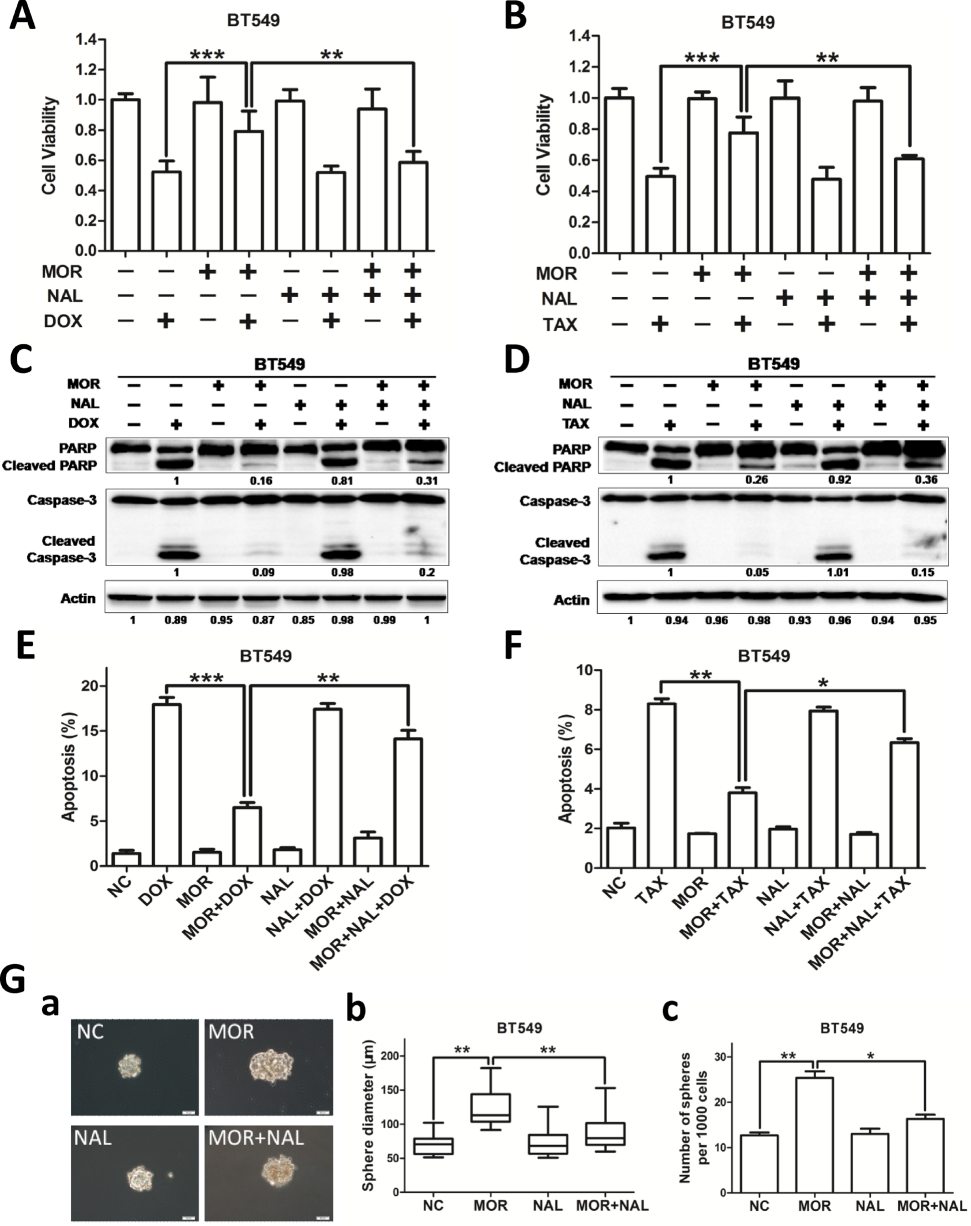
Nalmefene reverses morphine-induced cancer stem cell properties and chemoresistance BT549 cells were pretreated with the morphine (10 μM), nalmefene (10 μM) respectively and conjunctively for 4 days, then added doxorubicin (0.5 μM) or paclitaxel (10 nM) for other two days. **(A–B)** Cell viabilities were measured by MTT analysis. **(C–D)** Cleavaged caspase-3 and PARP were detected by western blotting. **(E–F)** Cell apoptosis was measured by Annexin V/PI co-staining assay. **(G)** a Sphere formation assay of BT549 cells with the same treatment (Scale bars, 50 μm). b–c Bar diagrams showed the diameter and number of mammospheres (spheres > 50 μm). *P < 0.05, **P < 0.01, ***P < 0.001. Error bars represent mean ± SD of triplicates.

To extend our observations, we further used nalmefene, an antagonist of morphine, to test whether it could reverse the functions of morphine in chemoresistance. Nalmefene alone had no significant effect on cell proliferation, and combination with morphine and nalmefene did not rescue the cell death caused by doxorubicin or paclitaxel (Figure 5A–5B). Compared to morphine with doxorubicin or paclitaxel, combination with morphine, nalmefene and doxorbicin (or paclitaxel) increased the cleavage of PARP and caspase-3. In contrast, combining morphine with anticancer drugs decreased the cleavage of PARP and caspase-3 in comparison to doxorubicin or paclitaxel alone (Figure 5C–5D). Moreover, compared with morphine alone, nalmefene plus morphine increased the apoptosis rate caused by doxorubicin or paclitaxel (Figure 5E–5F). Furthermore, nalmefene alone did not have significant effect on the sphereformation ability. However, treatment with nalmefene and morphine resulted in a decreased sphere numbers and diameters compared with morphine alone (Figure 5G). Taken together, these results indicate that nalmefene could reverse the effect of morphine in both sphere forming ability and chemoresistance.

### Nalmefene reverses morphine-increased tumorigenesis

**Figure 6:**
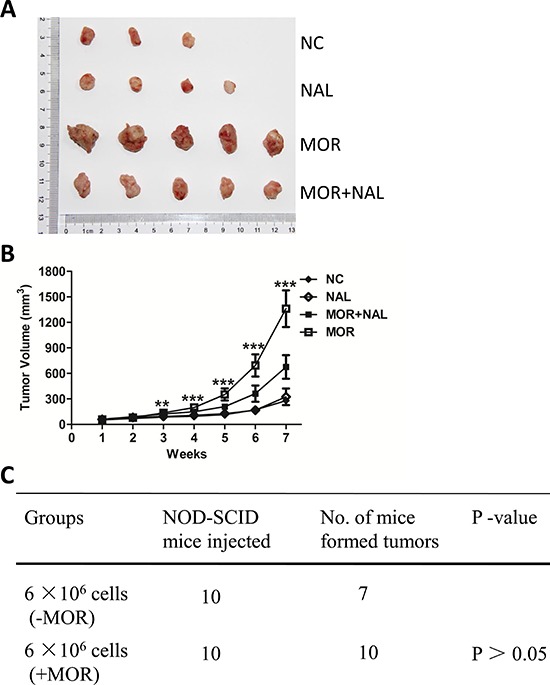
Nalmefene reverses morphine-increased tumorigenesis **(A)** Representative images of subcutaneous tumors taken on day 42 (*n* = 5). **(B)** The subcutaneous tumor growth curves of harvested tumors (*n* = 5). Data show the mean tumor volume ± SD Asterisks were compared morphine group to morphine plus nalmefene group (*P < 0.05, **P < 0.01, ***P < 0.001). **(C)** Summary of tumors xenograft formation of different treatment in NOD/SCID mice.

To investigate whether morphine could promote cancer development and the effect could be blocked by nalmefene *in vivo*, we established a NOD/SCID mouse model. BT549 cells were subcutaneously injected into mice and following treatment as mentioned. As we expected, no significant difference was found in tumor growth between the normal saline control group and the nalmefene group, which indicated that nalmefene had little effect on tumor growth. Compared with the normal saline group, morphine group showed a litter larger tumor volume on day 21 and a 2.1-fold larger tumor volume on day 36 (Figure 6A–6B). In contrast, the morphine plus nalmefene group demonstrated a decrease of tumor growth on day 24 and a 2.0-fold smaller tumor volume on day 42 in comparison to the morphine group (Figure 6A–6B). All the mice injected with morphine (all of 10) formed tumors, while 3 of 10 mice injected with saline or nalmefene failed to form tumors (Figure 6C). These results indicate that morphine promotes tumorigenesis which can be reversed by nalmefene.

## DISCUSSION

Most breast cancer patients use morphine to relieve severe pain. However, morphine contributes to the proliferation, invasion and metastasis of cancer cells, suggesting that long-term using morphine may render some side-effect on breast cancer patients who receive chemotherapy. In this study, we find that morphine could increase mammosphere forming ability and enrich cancer stem cell population of MCF-7, BT549 and MCF-10A cells (Figure 1), accompanies with increasing expression of stem cell related genes in human breast cancer cells (Figure 2). Meanwhile, morphine promotes EMT and metastasis in breast cancer cells (Figure 3). In addition, long-time using morphine leads to resistance of breast cancer cells to paclitaxel and doxorubicin (Figure 4). Furthermore, nalmefene could reverse the effect of morphine on breast cancer stem cell proportion, chemoresistance (Figure 5) and tumorigenesis (Figure 6).

A previous study showed that morphine displayed anticancer activity and inhibited the activation of nuclear transcription factor ĸB (NF-ĸB) [32]. Morphine decreases cell proliferation in many human cancer cells including breast cancer, lung cancer, gastric cancer and prostate cancer [33–36]. Moreover, morphine induces G_1_ cell cycle arrest and apoptosis by stimulating the phosphorylation of p53 at Ser^9^ and/or Ser^15^ [37]. Furthermore, long-term using of morphine to mice with transplanted neuroblastoma tumors inhibits tumor growth and prolongs the survival time, which can be abolished by morphine antagonist, naloxone [38, 39]. We speculated that the discrepancies might be resulted from different morphine doses used, route of administration, and/or plasma doses achieved at steady state.

Numerous studies have indicated that acquired drug resistance could attribute to the presence of CSCs [30, 40]. For instance, phosphorylated STAT3 promoted the stem-like cell phenotype in HER2-overexpressing breast cancer cells, which was resistant to Herceptin [41]. Similarly, miR125 maintained cancer stem-like side population fraction, which was higher expression in chemotherapy resistant patients than in chemotherapy responsive patients [42]. In consistent, our study showed morphine promoted the stem-like cell phenotype and induced EMT, which could contribute to the chemoresistance.

Stem cell markers Sox2, Oct4 and Nanog play pivotal roles in cancer development and drug resistance [43]. Our findings show that morphine significantly increased these stem cell markers supporting the notion that long-time using morphine leads to enrichment of stem cell properties and drug resistance.

EMT, characterized by the loss of epithelial differentiation and gain of mesenchymal phenotype, accompanied with metastasis and drug resistance, is tightly linked with the biology of CSCs [44]. Recent studies have demonstrated that the induction of immortalized human mammary epithelial cells into mesenchymal phenotype, resulting in the loss of epithelial phenotype and the acquisition of mesenchymal phenotype, concomitant with the acquisition of CD44^+^/CD24^−/low^ expression pattern [45]. Another report observed that the induction of EMT by CD8^+^ T cells led to the outgrowth of tumor *in vivo*. Interestingly, the mesenchymal tumor cells with a CD44^+^/CD24^−/low^ phenotype could re-establish an epithelial tumor and exhibit drug resistance, which resembled the characteristics of breast CSCs [46]. In our study, morphine induced EMT by elevating the expression of nuclear β-catenin and promoted metastasis in breast cancer cells. Taken together, these reports and our study strongly suggest that the induction of EMT could generate stem-like cells.

Nalmefene, an opiate derivative, is similar to the opiate antagonist naltrexone in structure and activity, which targets mu-, delta-, and kappa-binding sites [47–49]. In the present study, we found that nalmefene could reverse the effect of morphine in promoting breast cancer stemness and chemoresistance of doxorubicin and paclitaxel. In our study, nalmefene (10 μM) alone had no effect on cell growth, apoptosis and sphere-forming ability (Figure 5). Moreover, preclinical and clinical studies have demonstrated that co-treatment with extremely low doses of opioid receptor antagonists can markedly enhance the efficacy and specificity of morphine and simultaneously attenuate opioid tolerance and dependence [50]. Thus, combination with morphine and low dosage of nalmefene would be more effective in cancer treatment without increasing drug resistance and aggressiveness.

In conclusion, we have uncovered the direct effects of morphine on inducing cancer stem cell properties and chemoresistance in breast cancer. Our data demonstrated that morphine led to chemoresistance of doxorubicin and paclitaxel. Thus, we suggest that combination with morphine and nalmefene improve the effectiveness of anti-tumour therapies. However, further clinical studies are needed to extend our observations and the potential mechanism is under pursuing in our lab.

## MATERIALS AND METHODS

### Cell lines and cell culture

The human breast cancer cell lines MCF-7, BT549 and the immortalized breast epithelial cell MCF-10A were obtained from the American Type Culture Collection (ATCC). The cell lines were authenticated at ATCC before purchase by their standard short tandem repeat DNA typing methodology. MCF-7 and BT549 cells were routinely maintained in high-glucose DMEM (Gibco) and RPMI1640 (Gibco) supplemented with 10% FBS, respectively. MCF-10A cell was cultured in DMEM/F12 medium (Invitrogen) supplemented with 5% (v/v) horse serum (HS, HyClone), 20 ng/ml EGF, 100 ng/ml cholera toxin, 0.01 mg/ml insulin and 500 ng/ml hydrocortisone.

### Drugs and reagents

Morphine hydrochloride was from Northeast Pharmaceutical Group (China). Nalmefene hydrochloride was from Haisike (China). Hoechst 33342 (Sigma) was dissolved in dH_2_0 to a stock concentration of 5 mg/mL and stored at at −20°C. Fumitremorgin C (FTC) purchased from Sigma was dissolved in DMSO to a stock concentration of 2 mM and stored at −20°C. Doxrubicin was purchased from KeyGene (China). Paclitaxel (Cell Signaling Technology) was dissolved in dimethyl sulfoxide (DMSO) to a stock concentration of 1 mM and stored at −20°C.

### MTT assay

MTT (Sigma) assay was used to assess the growth of breast cancer cells. Cells (2.5–5 × 10^3^) were plated in 96-well flat bottom plates in a final volume of 200 μl. When attached to the flat, cells were exposed to drugs for 24 to 48 hours. Cell survival was assessed as described previously [15].

### Immunofluorescence staining

Immunofluorescence staining of cells was performed as previously described [16]. Briefly, cells were fixed in 4% para-formaldehyde-PBS at room temperature for 20 minutes and permeabilized in 0.5% Triton X-100 in PBS for 10 minutes at 4°C. Cells were then blocked with 3% BSA and incubated with primary antibody against E-cadherin (Cell Signaling Technology), N-cadherin (Abcam) and β-catenin (Millipore) followed by a FITC conjugated second antibody (Invitrogen), counterstained with DAPI (1 μg/ml) and visualized using a confocal microscope (Leica).

### Transwell migration and invasion assays

For migration assay, cells (5 × 10^4^) pretreated with morphine (0, 1, 10 μM) for 4 days were resuspended in culture medium with the same concentration of morphine and placed into uncoated membrane in the upper chamber (24-well insert, 8 μm, Corning Costar). DMEM supplemented with 10% FBS was used as an attractant in the lower chamber. After being incubated for 24 hours, cells migrated through the membrane were fixed with 4% paraformaldehyle (Santa Cruz) and stained with 1% crystal violet (Shanghai Sangon Company). The stained cell images were captured by microscope (Olympus), and five random fields at 10× magnification were counted. Results represented the average of triplicate samples from three independent experiments.

For invasion assays, cells (8 × 10^4^) were placed into 50 μl matrigel (BD Biosciences) coated membrane in upper chamber and being incubated for 36 hours. Following steps were similar with migration assays.

### Apoptosis assay

Cells were treated with the indicated concentrations of morphine, doxorubicin (or paclitaxel) and nalmefene, followed by staining with Annexin V and PI for 15 minutes at 4°C in the dark. Apoptotic cells were determined by the Annexin V/FITC apoptosis detection kit (KeyGen KGA108, China) and an Accuri C6 flow cytometer according to the manufacturer's instructions.

### Detection of breast cancer surface marker CD44/CD24

To evaluate CD24 and CD44 expression, cells were cultured with morphine (10 μM) for 14 days. Cells (2 × 10^6^) were harvested and incubated with antibodies against CD44 FITC (BD Pharmingen) and CD24 PerCP-Cy5.5 (BD Pharmingen) for 30 minutes at 4°C in the dark. Unbound antibody was washed away through two cycles of washing with PBS. Then cells were analyzed on a BD FACS Calibur flow cytometer. These data were analyzed by Cellquest Pro and at least 20,000 events per sample were collected.

### Side population assay

MCF-7 cells (10^6^/ml) were incubated with 2 μM FTC, ABCG2-specific inhibitor, for 20 minutes in negative control tubes before adding Hoechst 33342. Then MCF-7 cells were incubated in Hanks' Balanced Salt Solutions (HBSS) supplemented with 2% FBS, 10 mM HEPES, 5 μg/ml Hoechst 33342 for 90 minutes at 37°C with intermittent mixing, followed by washing with cold medium. Cells were resuspended at 2×10^6^ cells/ml. PI was added to a final concentration of 2 μg/ml to discriminate dead cells. The gating of side population was based on negative controls in which FTC was used. Then cells were analyzed on a BD FACScalibur flow cytometer. At least 100,000 events per sample were collected for data analysis.

### Quantitative reverse transcriptase polymerase chain reaction

Total RNA was extracted by using TRIzol reagent (Invitrogen), which was used to generate cDNA by using SuperScript III RT (Invitrogen) with an oligo-dT primer. Q-PCR was performed using Platinum SYBR Green qPCR SuperMix (Invitrogen) as recommended by the manufacturer. The primers used were listed in Supplementary Table 1. ACTB was used as the internal control.

### Sphere formation assay

Sphere formation was performed in ultralow attachment plates (Corning) with medium supplemented with 2% B27, 20 ng/ml bFGF, and 20 ng/ml EGF. BT549 and MCF7 cells were planted at the density around 2 cells/μl and cultured at 37°C in 5% CO_2_. After 14 days, the spheres greater than 50 μm diameter were counted at 40 x magnification under Olympus microscope. Sphere formation efficiency (SFE) = Number of spheres per 1000 cells.

### Western blot

Cells (1 × 10^6^) were washed with ice-cold PBS and lysed in RIPA lysis buffer on ice. Cells (1 × 10^7^) were subjected for nuclear/cytoplasmic protein isolation by using Nuclear/Cytosol Fractionation Kit (Biovision K266–25). Equal amounts of proteins were separated by SDS-PAGE and transferred to a NC membrane. The membranes were blocked with 5% fat-free milk in TBST at room temperature for 1 hour and probed with primary antibodies against Oct4, PARP, caspase-3 (Cell Signaling Technology), Nanog (Abcam), Sox2 (Santa Cruz), E-cadherin (Epitomics), N-cadherin (Abcam), β-actin (Proteintech), Lamin B1 (Epitomics) and β-catenin (Millipore) at 4°C overnight, followed by incubation with appropriate secondary antibodies (Thermo) at room temperature for 60 minutes. Antibody binding was detected with an enhanced chemiluminescence kit (Amersham, UK). Relative quantities were indicated by software Image Lab 4.0.1 (Bio-Rad Company).

### Mouse xenograft assay

BT549 cells (6 × 10^6^) in 100 μl were subcutaneously injected at the right dorsal flank of female mice (4–6 week, 18–25 g, Dalian, China). Mice were treated subcutaneously every day with saline (*n* = 5), nalmefene (*n* = 5), morphine (*n* = 5), or nalmefene plus morphine (*n* = 5) right after tumor cell implantation. Due to the potential desensitization of opioid receptors, the dose of morphine and nalmefene were increased stepwise (5, 10 and 15 mg/kg s.c. for every two weeks). For drug combination, the nalmefene dose was one-tenth of the morphine dose because this ratio is generally considered to result in a complete antagonism of antinociceptive effects of morphine [17]. The body weight of the animals and the two perpendicular diameters (a and b) were recorded every 3 days. Tumor volume (V) was calculated according to the following formula: V = (a*b*b)/2 [18]. Forty-two days after caudal intravenous injection, the mice were euthanized and dissected. The protocol was performed as previously described [19]. All animal procedures were approved by the Animal Ethics Committee of Dalian Medical University.

### Statistical analysis

Each experiment was performed in triplicate and repeated at least three times. The differences in mean values among groups were evaluated and expressed as the mean ± SD. A *P*-value less than 0.05 was considered statistically significant (*P < 0.05, **P < 0.01, ***P < 0.001). Student's *t*-test was used to compare the expressions of cell surface markers, side population analysis, cell viability, relative mRNA levels, migrated cells and invaded cells. Mann-Whitney test was used to compare the sphere volumes. The ANOVA test was used to compare the tumor volume.

## SUPPLEMENTARY FIGURE AND TABLE



## Abbreviations

BCSCs: Breast cancer stem cells
CSCs: Cancer stem cells
EMT: Epithelial to mesenchymal transition
FTC: Fumitremorgin C
MOR: Morphine
TAX: Paclitaxel
DOX: Doxorubicin
NAL: Nalmefene
PARP: Poly-ADP-ribose polymerase
PI: Propidium iodide
ABCG2: ATP-binding cassette sub-family G member 2
