# Correction: Morphine promotes cancer stem cell properties, contributing to chemoresistance in breast cancer

**DOI:** 10.18632/oncotarget.28907

**Published:** 2026-09-30

**Authors:** Dong-Ge Niu, Fei Peng, Wei Zhang, Zhong Guan, Hai-Dong Zhao, Jing-Lin Li, Kai-Li Wang, Ting-Ting Li, Yan Zhang, Fei-Meng Zheng, Fan Xu, Qian-Ni Han, Peng Gao, Qing-Ping Wen, Quentin Liu

**Affiliations:** ^1^Institute of Cancer Stem Cell, Dalian Medical University, Dalian 116044, China; ^2^Sun Yat-sen University Cancer Center, State Key Laboratory of Oncology in South China, Collaborative Innovation Center of Cancer Medicine, Guangzhou 510060, China; ^3^Department of Anesthesia, The First Affiliated Hospital, Dalian Medical University, Dalian 116044, China; ^4^Department of Otorhinolaryngology, Sun Yat-sen Memorial Hospital, Sun Yat-sen University, Guangzhou 510120, China; ^5^Department of Breast Surgery, The Second Affiliated Hospital of Dalian Medical University, Dalian 116023, China; ^6^Department of Clinical Pharmacology, College of Pharmacy, Dalian Medical University, Dalian 116044, China; ^*^These authors have contributed equally to this work

**This article has been corrected:** Oncotarget has concluded its investigation of this paper. It was found that Figure 3E contains a duplication of β-catenin immunofluorescence images of MCF-7 cells treated with morphine (MOR) at 1 and 10 μM, and Figure 3F contains a duplication of E-cadherin at 1 μM and β-catenin at 0 μM MOR images of BT549 cells. The authors confirmed the accidental misplacement of images, provided the original data and replaced the β-catenin image for MCF-7/1 μM MOR in Figure 3E (bottom middle panel) and the β-catenin image for BT549/0 μM MOR in Figure 3F (bottom left panel). The corrected Figure 3 is provided below.

The authors would also like to address a question raised by Dr. Amanda Capes-Davis regarding whether mycoplasma contamination or cross-contamination during cell passage could influence the effects of morphine. The authors stated: “We performed standard short tandem repeat (STR) DNA typing methodology to re-authenticate three cell lines at the end of experimental works in Beijing Microread Genetics Co., Ltd, China. Mycoplasma testing of cell lines was also performed during cell culture by PCR in our lab. These detections ensured that our cell lines used in the paper had no mycoplasma contamination or cross-contamination.” The authors submitted the official testing results and the updated text for the “Cell lines and cell culture” section of the Materials and Methods, provided below.

All authors agree with these corrections, state that they do not alter the article’s conclusions, and sincerely apologize for any inconvenience caused to readers.

## MATERIALS AND METHODS

### Cell lines and cell culture

The human breast cancer cell lines MCF-7, BT549 and the immortalized breast epithelial cell MCF-10A were obtained from the American Type Culture Collection (ATCC). The cell lines were authenticated at ATCC before purchase and were authenticated in Beijing Microread Genetics Co., Ltd, China at the end of experimental work by standard short tandem repeat (STR) DNA typing methodology. MCF-7 and BT549 cells were routinely maintained in high-glucose DMEM (Gibco) and RPMI1640 (Gibco) supplemented with 10% FBS, respectively. MCF-10A cell was cultured in DMEM/F12 medium (Invitrogen) supplemented with 5% (v/v) horse serum (HS, HyClone), 20 ng/ml EGF, 100 ng/ml cholera toxin, 0.01 mg/ml insulin and 500 ng/ml hydrocortisone. Mycoplasma testing of cell lines was performed during cell culture by PCR.

Original article: Oncotarget. 2015; 6:3963–3976. 3963-3976. https://doi.org/10.18632/oncotarget.2894

**Figure 3:**
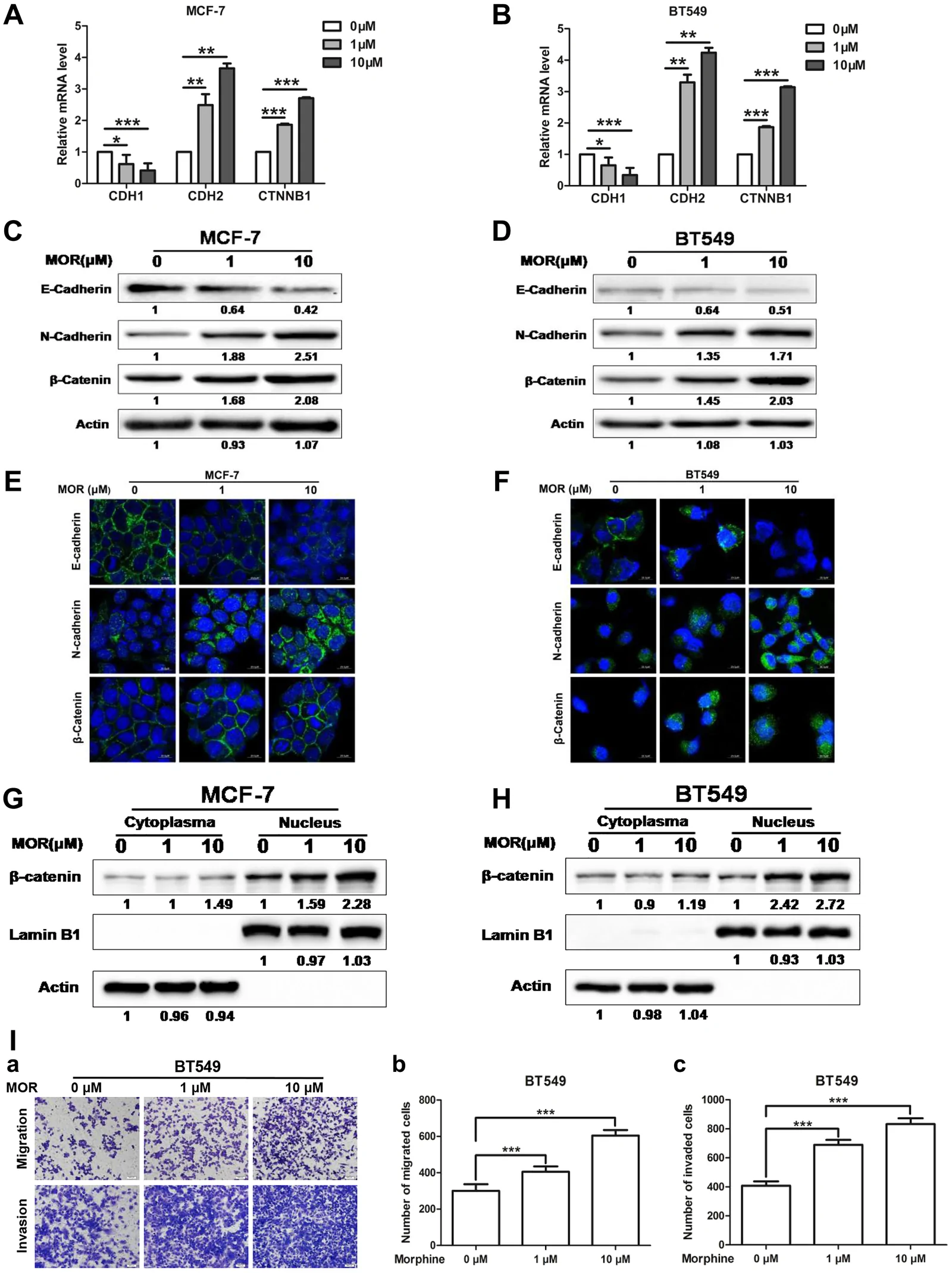
Morphine promotes EMT and metastasis. MCF-7 and BT549 cells were treated with morphine (0, 1, 10 μM) for 4 days. (**A**, **B**) The mRNA levels of CDH1, CDH2 and CTNNB1 were measured by Q-PCR. ^*^P < 0.05, ^**^P < 0.01, ^***^P < 0.001. Error bars represent mean ± SD of triplicates. (**C**, **D**) E-cadherin, N-cadherin and β-catenin levels of cell lysates were measured by western blotting analysis. (**E**, **F**) Immunofluorescence was performed using FITC-labeled phalloidin, E-cadherin, N-cadherin, β-catenin. Nuclei were stained with DAPI (Scale bar, 20 μm). (**G**, **H**) Cells were subjected to nuclear/cytoplasmic protein isolation. The expressions of β-catenin in nucleus and cytoplasm were determined by western blotting. Lamin B1 and β-actin were taken as nuclear and cytoplasmic control respectively. (**I**) (**a**) Representative images of migrated and invaded cells are shown. (**b**, **c**) Bar diagrams showed the results of migrated and invaded cells untreated and treated with morphine in BT549 cells as described. ^*^P < 0.05, ^**^P < 0.01, ^***^P < 0.001. Error bars represent mean ± SD of triplicates.

