# Supplementary material for: Morphine promotes cancer stem cell properties, contributing to chemoresistance in breast cancer
Source: Oncotarget. 2015 Feb 20;6(6):3963–76. doi: 10.18632/oncotarget.2894 (PMC4414166; doi:10.18632/oncotarget.2894)
Supplement: Supplementary file 1 [file oncotarget-06-3963-s001.pdf]

SUPPLEMENTARY FIGURE AND TABLE

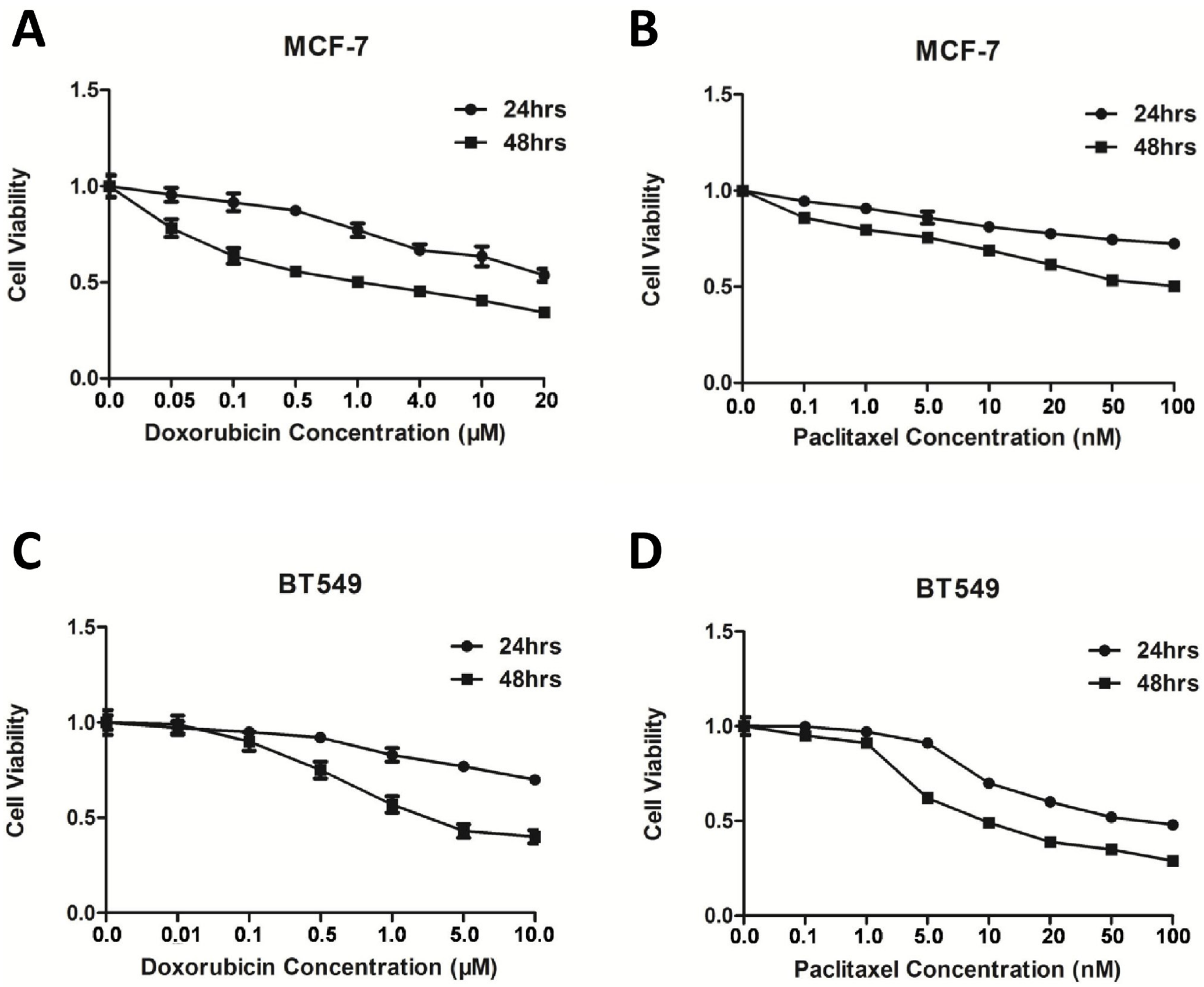

Supplemental Figure 1: Anticancer drugs inhibit proliferation in breast cancer cells. (A–B) Proliferation of MCF-7 cells treated with doxorubicin and paclitaxel were analyzed by MTT assay. **(C–D)** Proliferation of BT549 cells treated with doxorubicin and paclitaxel were analyzed by MTT assay. All experiments were performed in triplicate.

Supplementary Table 1: Primer sequences used in Q-PCR

mRNA	Primers	Sequences (5′–3′)
OCT4	Forward	agcgaaccagtatcgagaac
	Reverse	ttacagaaccacactcggac
SOX2	Forward	tcaggagttgtcaaggcagag
	Reverse	cgccgccgatgattgttatta
NANOG	Forward	cagccctgattcttccaccagtccc
	Reverse	tggaaggttcccagtcgggttcacc
ACTB	Forward	ttgccgacaggatgcagaagga
	Reverse	aggtggacagcgaggccaggat
CDH1	Forward	gtcactgacaccaacgataatcct
	Reverse	tttcagtgtggtgattacgacgtta
CDH2	Forward	ggacagttcctgagggatca
	Reverse	ggattgccttccatgtctgt
CTNNB1	Forward	tctcctcagatggtgtctgct
	Reverse	tgaaccaagcattttcaccag
